# Machine learning-derived R-loop-related diagnostic signature for psoriasis: a multi-cohort and single-cell validation study

**DOI:** 10.3389/fimmu.2026.1839925

**Published:** 2026-10-05

**Authors:** Yeqi Feng, Lebin Song, Xiaozheng Jiang, Hongyu Ruan, Jiayi Zhao, Yan Lu

**Affiliations:** Department of Dermatology, The First Affiliated Hospital of Nanjing Medical University, Nanjing, China

**Keywords:** keratinocyte proliferation, machine learning, psoriasis, R-loop, SRPK1

## Abstract

**Background:**

Psoriasis is a chronic inflammatory skin disease characterized by dysregulated immune responses and keratinocyte (KC) hyperproliferation. Although therapeutic strategies have advanced considerably, achieving durable long-term remission remains challenging. R-loops are three-stranded nucleic acid structures involved in genomic stability and inflammatory regulation in multiple diseases; however, their role in psoriasis remains poorly understood.

**Objective:**

To investigate the involvement of R-loops in psoriasis, develop an R-loop-related diagnostic signature, and identify key molecular drivers of disease pathogenesis.

**Methods:**

Transcriptomic data from five public bulk RNA expression cohorts (GSE30999, GSE41664, GSE13355, GSE6710, and GSE14905) and one single-cell RNA sequencing (scRNA-seq) dataset (GSE173706) were integrated for analysis of psoriatic and non-lesional skin samples. R-loop-related differentially expressed genes were identified and used to construct an R-loop-related diagnostic signature (RRDS) using the Elastic Net machine learning algorithm. Model performance was evaluated using three complementary analytical approaches, and SHapley Additive exPlanations (SHAP) analysis was performed to quantify the contribution of individual genes. scRNA-seq data were further analyzed to validate the findings at the cellular level. In addition, the biological function of SRPK1 was examined in HaCaT cells by siRNA-mediated knockdown, quantitative real-time PCR (qRT-PCR), and Cell Counting Kit-8 (CCK-8) assays.

**Results:**

R-loop activity was significantly elevated in psoriatic lesions compared with non-lesional skin. We established and validated an eight-gene RRDS that accurately distinguished psoriatic from healthy skin. SHAP analysis identified SRPK1 as the most influential feature in the diagnostic model. Single-cell analysis further localized SRPK1 to a pathogenic KC_4 subpopulation at a critical stage of keratinocyte differentiation. Functional validation in HaCaT cells confirmed efficient SRPK1 knockdown by qRT-PCR and demonstrated that SRPK1 silencing significantly suppressed keratinocyte proliferation over 96 hours, as determined by CCK-8 assays (P < 0.001).

**Conclusion:**

This study provides the first evidence linking R-loop dysregulation to psoriasis. We developed a R-loop-related diagnostic signature and identified SRPK1 as a potential key driver of psoriasis pathogenesis. Together with the *in vitro* findings, these results suggest that SRPK1 may serve as both a promising diagnostic biomarker and a potential therapeutic target in psoriasis.

## Introduction

Psoriasis is a chronic, immune-mediated inflammatory skin disease driven by a complex interplay between keratinocytes and immune cells. Its pathogenesis is centered on dysregulated cytokine networks, particularly the TNF-α/IL-23/IL-17 axis, which promotes epidermal hyperproliferation, aberrant keratinocyte differentiation, and sustained inflammatory responses. In addition to cutaneous manifestations, psoriasis is increasingly recognized as a systemic disorder, and achieving long-term remission remains a major clinical challenge primarily owing to high relapse rates following treatment discontinuation (1). Recent advances in molecular immunology and translational research have considerably refined our understanding of these pathogenic circuits.

Therapeutically, the management of psoriasis has evolved from conventional systemic agents toward targeted biologic therapies and small-molecule inhibitors. Biologics directed against TNF-α, IL-17, and IL-23 have dramatically improved clinical outcomes and have become central to the management of psoriasis because of their high efficacy and favorable long-term disease control. Notably, secukinumab, an anti-IL-17A monoclonal antibody, has demonstrated robust clinical, histologic, and molecular efficacy, further underscoring the pivotal role of cytokine-targeted intervention in psoriasis treatment (2). In parallel, recent work has highlighted that psoriasis should be viewed not only as an immune-mediated inflammatory dermatosis but also as a disease with dynamic immune plasticity and precision-treatment implications, reinforcing the clinical importance of mechanistic studies that may support individualized therapeutic strategies (3).

The immunoinflammatory process in psoriasis is understood to be mediated by various immune cells, such as dendritic cells, Th17 cells, and resident memory T cells (4–6). These cells produce Th1- and Th17-associated cytokines that establish a crosstalk with KCs, driving KCs’ aberrant proliferation and differentiation. Furthermore, emerging evidence indicates that KCs are not merely passive targets of cytokines; they actively participate in the pathogenesis of psoriasis (7). Specifically, KCs secrete cytokines such as IL-17 and IL-36, which in turn act upon them ​autocrinally. Under stimulation by pro-inflammatory factors, KCs are induced for IL-36 (8). This in turn, stimulates KCs to generate more TNF-α and IL-17C (9). This establishes a potent inflammatory loop (10). Thus, KCs function as both the source and target of IL-36 and IL-17C, establishing a positive feedback loop that further promotes psoriatic inflammation. Consequently, the level of inflammation in KCs is of crucial importance.

An R-loop is a three-stranded nucleic acid structure. It is formed by an RNA-DNA hybrid and a displaced single-stranded DNA (11). Under physiological conditions, genome-wide data indicate that R-loops are extensively distributed throughout the genome, where they participate in key biological processes such as gene expression regulation, DNA replication, and epigenetic modification (12). Conversely, the excessive accumulation of R-loops can induce DNA double-strand breaks, thereby compromising genomic stability (13). This instability, in turn, can trigger innate immune responses and promote the release of inflammatory factors by activating inflammatory cascades through signaling pathways such as cGAS-STING and NF-κB (14).

Dysregulation of these processes is linked to several diseases, especially cancers and inflammatory diseases. For instance, R-loops drive tumor progression by promoting inflammatory signaling cascades, and in lung adenocarcinoma (LUAD), aberrant R-loop regulation has been linked to an inflammatory microenvironment (15). This link is also evident in UV-induced skin injury, where R-loop accumulation activates inflammatory gene expression, while inhibiting R-loops downregulates the expression of pro-inflammatory genes (16). Likewise, in inflammatory bowel disease, ​DHX9​ loss ​drives​ pathological R-loop accumulation, promoting innate immune activation through the cGAS-STING axis, ultimately promoting disease pathogenesis (17). Collectively, these studies demonstrate that dysregulated R-loop levels are directly associated with various inflammatory diseases, suggesting that targeting this mechanism may offer novel therapeutic strategies. Psoriasis, characterized by inflammation-driven cellular hyperproliferation, exhibits features of DNA replication stress and genomic instability, which commonly induced by R-loop dysregulation. To date, no investigations have examined​ the potential involvement of R-loops in psoriasis.

From a clinical perspective, robust molecular biomarkers may provide complementary value for disease assessment, particularly in situations where inflammatory skin lesions present with overlapping phenotypes or are complicated by drug-induced cutaneous eruptions (18). Building on the premise that R-loops are essential to the pathophysiology and mechanisms of inflammatory diseases, this study aimed to explore their role in psoriasis. We Investigate the impact of R-loop related genes on psoriasis pathophysiology using scRNA-seq and bulk transcriptomics. Utilizing ML algorithms, we constructed an R-loop-related diagnostic signature (RRDS) and identified eight genes significantly associated with psoriasis prognosis, with serine-arginine protein kinase 1 (SRPK1) emerging as the gene with the most substantial positive contribution. Furthermore, scRNA-seq analysis revealed that SRPK1 expression was significantly upregulated in a specific KC subpopulation (KC_4), where it appeared to modulate multiple signal transduction pathways. This finding suggests a close association between R-loops and the pathological processes of psoriasis. To our knowledge, this study is the first to explicitly define the relationship between R-loop related gene expression and the pathological progression of psoriasis, providing compelling evidence that R-loop related genes may serve as novel biomarkers for precise diagnosis and as potential therapeutic targets for psoriasis.

## Materials and methods

### Data collection and preprocessing

Publicly available psoriasis-related bulk transcriptome datasets were downloaded from the Gene Expression Omnibus (GEO) database, including GSE30999 (n = 170), GSE41664 (n = 157), GSE13355 (n = 116), GSE14905 (n = 58), and GSE6710 (n = 26). These datasets contained psoriatic lesional (LS) and non-lesional (NL) skin samples. The human psoriasis scRNA-seq dataset (GSE173706) for single-cell analysis was also obtained from the GEO database. The dataset included 11 perilesional (PN) and 14 psoriatic plaque (PP) skin samples. **Supplementary Table 1**​ summarizes all datasets employed in this investigation. The R-loop related genes was obtained from the R-loopBase database (19). Based on this database, A total of​ 1185 R-loop related genes in humans were identified from the database (**Supplementary Table 2**).

Expression matrices were processed in R software. Probe identifiers were converted to official gene symbols according to the corresponding platform annotation files. Probes mapped to multiple genes were removed, and when multiple probes corresponded to the same gene symbol, the average expression value was retained. Data were log2-transformed when necessary before downstream analyses. For analyses involving combined bulk cohorts, only shared genes across datasets were retained, and inter-dataset batch effects were corrected using the ComBat algorithm implemented in the “sva” R package.

### Differential expression analysis and functional enrichment analysis

Differential expression analysis was performed separately in each bulk dataset using the “limma” R package. Genes with an absolute log2 fold change (|log2FC|) > 1 and P < 0.05 were considered differentially expressed genes (DEGs). Consensus DEGs were obtained by separately intersecting the upregulated and downregulated DEG lists from the five bulk cohorts using the same threshold (|log2FC| > 1 and P < 0.05). The resulting consensus upregulated and consensus downregulated genes were then combined to generate the final consensus DEG set. Subsequently, this set of final consensus DEGs was intersected with the R-loop related genes set to obtain the R-loop-related DEGs for subsequent analysis.

Functional enrichment analyses, including Gene Ontology (GO) and the Kyoto Encyclopedia of Genes and Genomes (KEGG) pathways, were performed for common DEGs using the R package clusterProfiler (version 4.12.6), with P-value < 0.05 was considered statistically significant. Protein-protein interaction (PPI) networks were constructed via the STRING database (v11.5) and set a confidence score > 0.4.

### Identification of top contributing genes via SHAP

To interpret the model and identify the most significant biomarkers, we computed SHapley Additive exPlanations (SHAP) values. The calculation was performed using the kernelshap R package (version 0.5.0), which employs a kernel-weighted local regression to estimate the contribution of each gene to the model output. Genes were ranked based on their mean absolute SHAP values, reflecting their global importance to the prediction.

### Machine learning algorithm for diagnostic model construction

We employed the Elastic Net logistic regression algorithm to build the diagnostic model. Elastic Net is a penalized regression method that linearly combines the L1 (Least Absolute Shrinkage and Selection Operator (LASSO)) and L2 (Ridge) penalties. This approach is particularly effective for high-dimensional biological data as it performs automated feature selection like LASSO, while also handling correlated predictors more effectively, a common characteristic of gene expression data.

The GSE30999 and GSE41664 datasets were designated as the training set, while GSE13355, GSE6710, and GSE14905 served as independent external validation sets. To correct for potential batch effects, we applied the ComBat function from the R package sva (version 3.40.0) to the expression data of the training cohort and the validation cohort separately. The model was trained on the expression data of R-loop-related DEGs from the training cohort. The gene expression values for each sample were standardized to eliminate the influence of different measurement scales.

The training was performed using the glmnet R package (version 4.1-8). The optimal value for the primary regularization parameter, lambda (λ), was identified through a 10-fold cross-validation procedure. The lambda.min value, which corresponds to the lambda that gives the minimum mean cross-validated error (deviance), was chosen to build the final model. An alpha of 0.5 was chosen to balance the penalties from LASSO (L1) and Ridge (L2) regression, which can be beneficial when there are correlated predictors. A random seed was set to ensure the reproducibility of the cross-validation and model-building process. The performance of the constructed Elastic Net model was rigorously evaluated. First, its predictive accuracy was assessed on the training dataset itself. Then, to confirm its robustness and generalizability, the model was applied to the independent external validation sets.

We computed a risk score for every sample in the training and validation cohorts by applying the predict function. The model’s discriminatory power was then evaluated by calculating the area under the receiver operating characteristic (ROC) curve (AUC) was ​computed​ employing the pROC package. An AUC value of 1.0 indicates a perfect classifier, while a value of 0.5 suggests no discriminative ability. The performance on both the training and validation sets was reported to assess the model’s generalizability and to check for potential overfitting. The clinical utility​ of the prediction models ​was assessed via​ decision curve analysis (DCA) implemented with the R package “ggDCA”.

### R-loop level scoring

To assess the potential R-loop formation capability in each sample, we established an R-loop scoring system based on the expression levels of R-loop-associated genes. First, a gene set characterizing R-loop activity was obtained from the R-loopBase database (19). Subsequently, Single-Sample Gene Set Enrichment Analysis (ssGSEA) was performed to quantify the enrichment score of this gene set for each individual sample. This analysis was conducted using the gsva function within the GSVA R package (version 1.40.1) with the method parameter set to ‘ssgsea’. The resulting score, termed the “R-loop score” reflects the collective expression level of R-loop regulators, where a higher score indicates increased R-loop pathway activity. Differences in R-loop scores between groups were evaluated using the Wilcoxon rank-sum test, with statistical significance defined as *P* < 0.05.

### scRNA-seq data analysis and pseudotime trajectory analysis

The GSE173706 dataset was analyzed under the Seurat R package (version 5.0.3). After filtration of​ cells containing either fewer than 200 or more than 4000 detected genes, or where mitochondrial genes constituted over 10% of the total, normalization was performed via the NormalizeDatafunction. Identification of highly variable genes was then conducted using FindVariableFeatures. To correct batch effects between samples, the Harmony algorithm was applied via the IntegrateLayers function. Following dimensionality reduction, we performed​ unsupervised clustering with the FindNeighborsand FindClustersfunctions. Cell populations were then annotated according to established marker gene expression and projected onto a UMAP plot for visualization. The expression pattern of SRPK1 was analyzed by comparing its expression levels across different cell types and between PN and PP samples. KCs were re-clustered to identify their internal subpopulations. GO functional enrichment analysis was conducted on the genes of specific KC subpopulations using enrichGO and compareCluster functions in the clusterProfiler package (version 4.12.6).

To infer the cellular lineage and pseudotime of KC subpopulations, trajectory analysis was performed using the Slingshot R package (version 2.7.0). This method identifies lineage structures through a cluster-based minimum spanning tree (MST) and fits principal curves to model these lineages. We obtained smooth trajectory curves by getCurves function.

### Cell culture and transfection

The human immortalized keratinocyte cell line HaCaT present in this study were obtained from the Cell Resource Center, Shanghai Institutes for Biological Sciences, Chinese Academy of Sciences (Shanghai, China). HaCaT cells were cultured in Dulbecco’s Modified Eagle Medium (DMEM) supplemented with 10% fetal bovine serum (FBS) and 1% penicillin-streptomycin at 37 °C in a humidified incubator with 5% CO^2^. SRPK1 knockdown was achieved using small interfering RNA (siRNA) synthesized by Hanbio Biotech (Shanghai, China). Cells were transfected with SRPK1-targeting siRNA using Lipofectamine 3000 (Invitrogen, Carlsbad, CA, USA) according to the manufacturer’s instructions. Knockdown efficiency was evaluated by qRT-PCR 48 h after transfection. The siRNA sequence is listed in **Supplementary Table 4**.

### RNA extraction and quantitative real-time PCR

Total RNA was extracted from cells using TRIzol reagent (Invitrogen, USA) according to the manufacturer’s instructions. cDNA was synthesized from 1 μg of total RNA using a reverse transcription kit (Vazyme, China). Quantitative real-time PCR (qRT-PCR) was performed using a SYBR Green-based PCR Master Mix on a real-time PCR detection system. The cycling conditions were as follows: initial denaturation at 95 °C for 30 s, followed by 40 cycles of 95 °C for 5 s and 60 °C for 30 s. Relative mRNA expression levels were calculated using the 2^-ΔΔCt^ method, with β-actin used as the internal control. Primer sequences are listed in **Supplementary Table 3**.

### Immunofluorescence data acquisition

Representative immunofluorescence images showing the subcellular localization of SRPK1 were obtained from the Human Protein Atlas (HPA) database (https://www.proteinatlas.org/), using antibody HPA056486 in the A-431 cell line. The images displayed triple staining for SRPK1 (green), alpha-Tubulin as a microtubule marker (red), and nuclei (blue, DAPI). Detailed experimental procedures are available on the HPA website.

### Cell proliferation assay

Cell proliferation was evaluated using the Cell Counting Kit-8 (CCK-8) assay. Transfected HaCaT cells (NC and KD groups) were seeded into 96-well plates at a density of 2 × 10^3^ cells per well. At specified time points (0, 24, 48, 72, and 96 hours), 10 μL of CCK-8 reagent was added to each well and incubated for 1 hours at 37 °C. The optical density (OD) at 450 nm was measured using a microplate reader.

### Statistical analysis

Unless otherwise specified, we conducted​ intergroup statistical comparisons by applying the ​Wilcoxon rank-sum test. A significance threshold of P < 0.05 was defined for all two-sided statistical tests. To account for multiple comparisons, the Benjamini-Hochberg procedure was applied to adjust the resulting P-values to control the false discovery rate (FDR). ​We performed​ all bioinformatic analyses with R software (version 4.4.0) and the aforementioned packages.

## Results

### Psoriatic lesions exhibit elevated R-loop activity compared to non-lesions

To assess R-loop expression in psoriasis, we first quantified R-loop related genes scores in five independent cohorts. Across all five independent datasets, the ssGSEA analysis revealed that R-loop activity was elevated in LS compared to NL samples (**Figure 1A**). As the volcano plots showing DEGs for each cohort (**Figure 1B**), we identified a consensus set of dysregulated genes. The Venn diagram further revealed this by showing the intersection of consensus upregulated and downregulated genes across all five cohorts. There are 758 consensus upregulated DEGs and 762 consensus downregulated DEGs. The intersection of the consensus DEGs and the R-loop related genes yielded the final list of “R-loop-related DEGs” for subsequent analysis. (**Figure 1C**).

**Figure 1:**
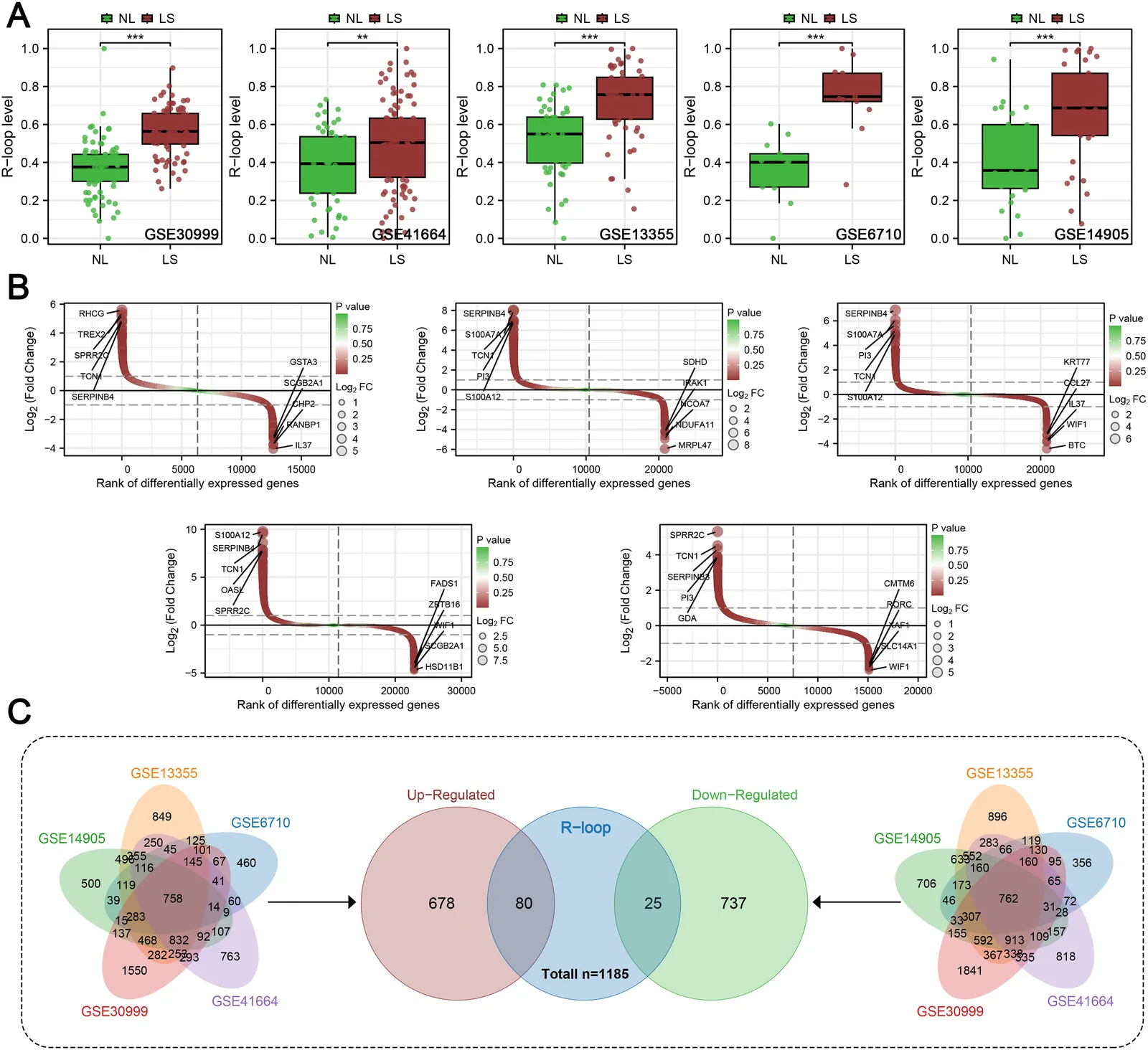
Elevated R-loop levels and identification of consensus DEGs in psoriatic lesions. **(A)** Box plot comparing R-loop scores between LS and NL based on ssGSEA algorithms across five independent datasets. **(B)** Volcano plot displays DEGs (|log2FC| > 1 and P < 0.05) from each of the five datasets. **(C)** Venn diagram showing the intersection of consensus upregulated and downregulated DEGs across all five datasets, and their overlap with the R-loop related genes. *LS, Lesional Skin; NL, Non-lesional Skin; ssGSEA, Single-Sample Gene Set Enrichment Analysis; DEGs, Differentially Expressed Genes.* Significance is denoted as *p < 0.05; **p < 0.01; ***p < 0.001.

### Functional analysis reveals a coordinated shift from cell adhesion to proliferation

To further understand the interplay between these proteins, PPI network analysis highlighted two distinct modules. Based on proteins like CDC45, MCMs and GINSs, the upregulated network was centered on cell cycle and DNA replication. However, the downregulated network was crucial for cell adhesion, cytoskeletal stability and protein homeostasis. This confirms a coordinated dysregulation of this opposing biological process, driving a shift away from a stable state towards active proliferation (**Figures 2A, B**). To reveal the change in biological process, we performed GO and KEGG on DEGs. Upregulated genes were significantly enriched in pathways governing cell cycle progression, DNA replication, and mitotic division. Conversely, downregulated genes were primarily associated with cell-substrate adhesion, focal adhesion signaling, and extracellular matrix organization (**Figures 2C–F**).

**Figure 2:**
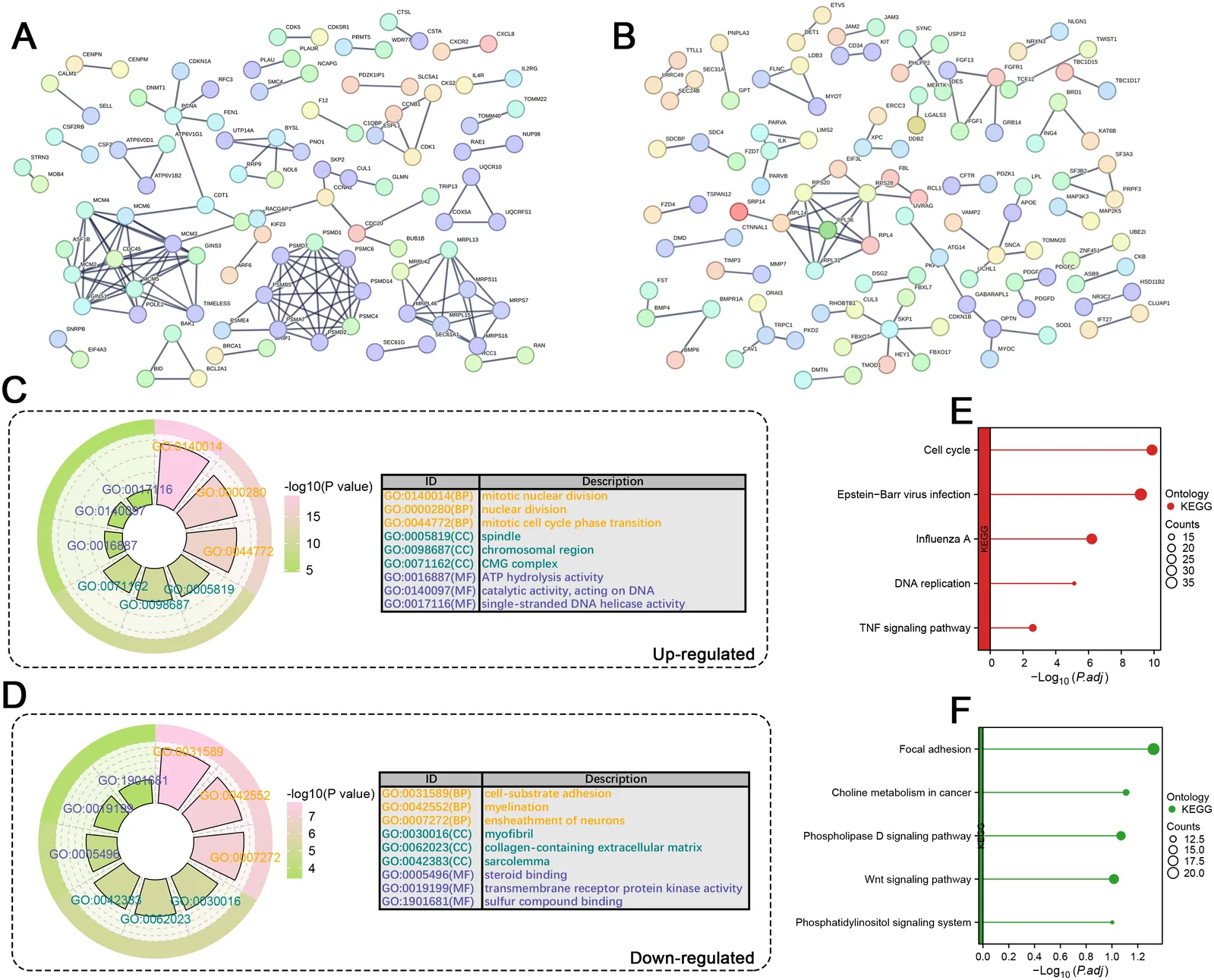
Functional enrichment and interaction network analysis of consensus DEGs. **(A)** PPI network of consensus upregulated DEGs, constructed using the STRING database. **(B)** PPI network of consensus downregulated DEGs. **(C)** GO enrichment analysis of consensus upregulated DEGs. **(D)** GO enrichment analysis of consensus downregulated DEGs. **(E)** KEGG pathway enrichment analysis of consensus upregulated DEGs. **(F)** KEGG pathway enrichment analysis of consensus downregulated DEGs. DEGs, Differentially Expressed Genes; PPI, Protein-Protein Interaction; GO, Gene Ontology; KEGG, Kyoto Encyclopedia of Genes and Genomes.

### Development of an RRDS and Identification of SRPK1 as a key driver

To develop a molecular diagnostic model for psoriasis, we first integrated two training cohorts (GSE30999, GSE41664) and three external validation sets (GSE13355, GSE6710, GSE14905) separately and then used the ComBat algorithm to correct for batch effects. Principal component analysis (PCA) confirmed that this method effectively removed inherent differences between the datasets (**Figures 3A, B**).

**Figure 3:**
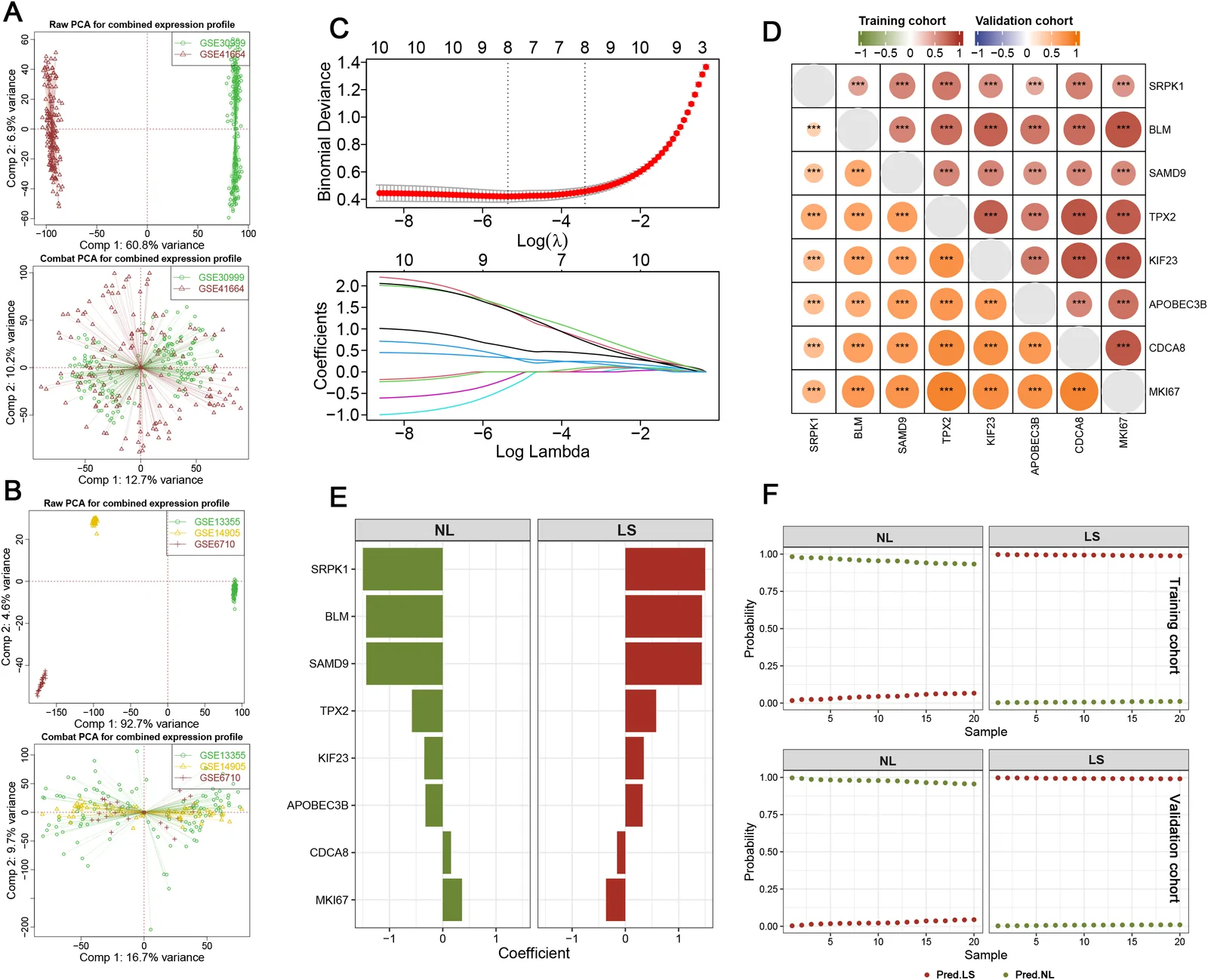
Construction and evaluation of the R-loop-related diagnostic signature (RRDS). **(A, B)** PCA plots of the merged expression profiles in the training cohort (**A**; GSE30999 and GSE41664) and validation cohort (**B**; GSE13355, GSE14905, and GSE6710), shown before and after batch-effect correction using the ComBat algorithm. **(C)** Feature selection using Elastic Net logistic regression. The upper panel shows the 10-fold cross-validation curve based on binomial deviance across different values of log(λ), with the numbers above indicating the number of non-zero coefficients. The lower panel shows the coefficient shrinkage paths of candidate genes as λ changes. The selected λ identified an 8-gene diagnostic signature. **(D)** Bubble correlation plot showing the pairwise co-expression relationships among the eight genes included in the RRDS in the training and validation cohorts. Color intensity indicates the strength and direction of correlation. **(E)** Bar plot showing the class-specific contributions of the eight genes in the final model for distinguishing LS samples from NL samples. **(F)** Prediction probability plots showing the performance of the RRDS model in classifying LS and NL samples in the training and validation cohorts. LS, Lesional Skin; NL, Non-lesional Skin; PCA, Principal Component Analysis; RRDS, R-loop-related Diagnostic Signature.

Furthermore, we applied the Elastic Net logistic regression algorithm to the adjusted training data to construct the diagnostic model. This method performs feature selection automatically during penalized regression. Through 10-fold cross-validation, we identified the optimal regularization parameter λ. The coefficient shrinkage path plot (**Figure 3C**) visually demonstrates how the model selected a minimal set of key genes from all R-loop-related DEGs, ultimately defining a concise 8-gene diagnostic signature termed the RRDS. The eight genes are: SRPK1, BLM, SAMD9, TPX2, KIF23, APOBEC3B, CDCA8, and MKI67. Correlation analysis revealed significant co-expression relationships among these genes (**Figure 3D**).

**Figure 3E** shows the class-specific contributions of each included gene to the classification of LS and NL samples. Among them, SRPK1 showed the largest contribution to the prediction of psoriasis. The model showed excellent classification performance on both the training set and external validation sets. As demonstrated by the prediction probability plots (**Figure 3F**), the model confirmed its accuracy and generalizability.

### Diagnostic performance and clinical utility of the RRDS

The diagnostic performance of the RRDS was rigorously evaluated. The combined 8-gene RiskScore demonstrated outstanding classification accuracy, achieving a high AUC in both the training cohort (**Figure 4C**) and the external validation cohorts (**Figure 4D**). While individual genes showed varied predictive power (**Figures 4A, B**), the combined signature was significantly more robust. Furthermore, the model’s clinical utility was confirmed. Calibration curves showed excellent agreement between the RiskScore’s predicted probabilities and the actual observed psoriasis cases in both training and validation sets (**Figures 4E,F**). Critically, DCA demonstrated that using the Risk Score provides a superior net benefit ​at a broad array of risk thresholds, supporting its potential clinical applicability (**Figures 4G, H**).

**Figure 4:**
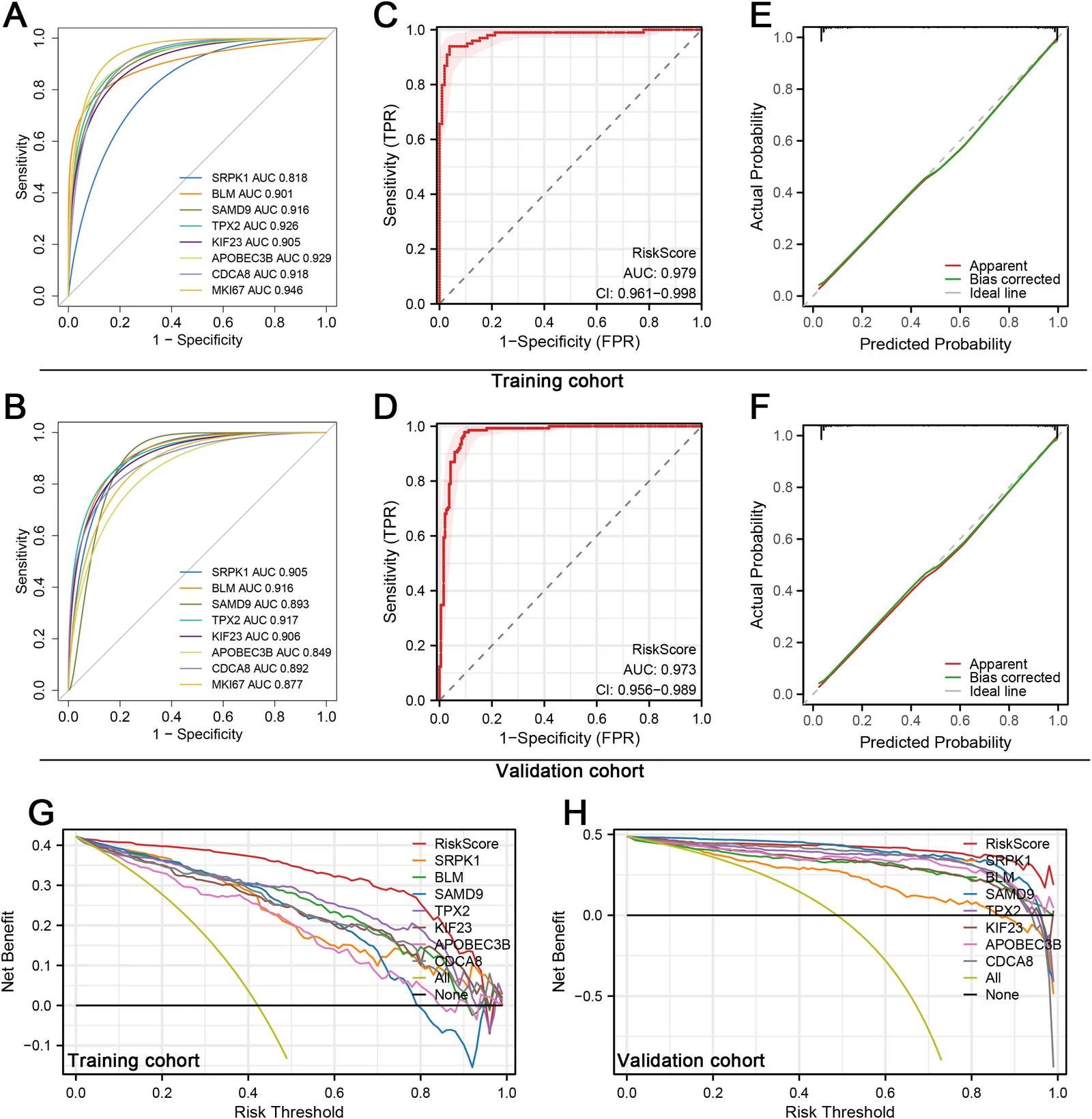
Diagnostic performance and clinical utility of the RRDS. **(A, B)** ROC curves showing the diagnostic performance of each individual gene in the RRDS within the training **(A)** and validation **(B)** cohorts. **(C, D)** ROC curves of the combined 8-gene RiskScore in the training **(C)** and validation **(D)** cohorts, showing significantly improved and robust diagnostic accuracy compared to individual genes. **(E, F)** Calibration curves assessing the agreement between the predicted probabilities of the RiskScore and the actual observed outcomes in the training cohort **(E)** and the validation cohort **(F)**. The dashed line represents perfect prediction. **(G, H)** DCA of the clinical utility of the RiskScore across different threshold probabilities in the training **(G)** and validation **(H)** cohorts. The RiskScore (red line) shows a higher net benefit than the “All” or “None” strategies over a wide risk threshold range.ROC, Receiver Operating Characteristic; AUC, Area Under the Curve; DCA, Decision Curve Analysis.

To better interpret the model’s decision-making process and quantify the specific contribution of each gene, we applied SHAP analysis. The SHAP feature importance bar plot (**Figure 5A**) clearly indicated that SRPK1 has a much higher mean absolute SHAP value than other genes, making it the most important feature. The SHAP summary plot (**Figure 5B**) further revealed the pattern of SRPK1’s influence: its high expression levels (red dots) consistently produced positive SHAP values, strongly pushing the prediction towards a psoriasis-positive outcome. This finding was consistently supported by the dependence plot (**Figure 5C**) and individual sample force plots (**Figure 5D**), confirming SRPK1 as the key driver gene in this diagnostic model.

**Figure 5:**
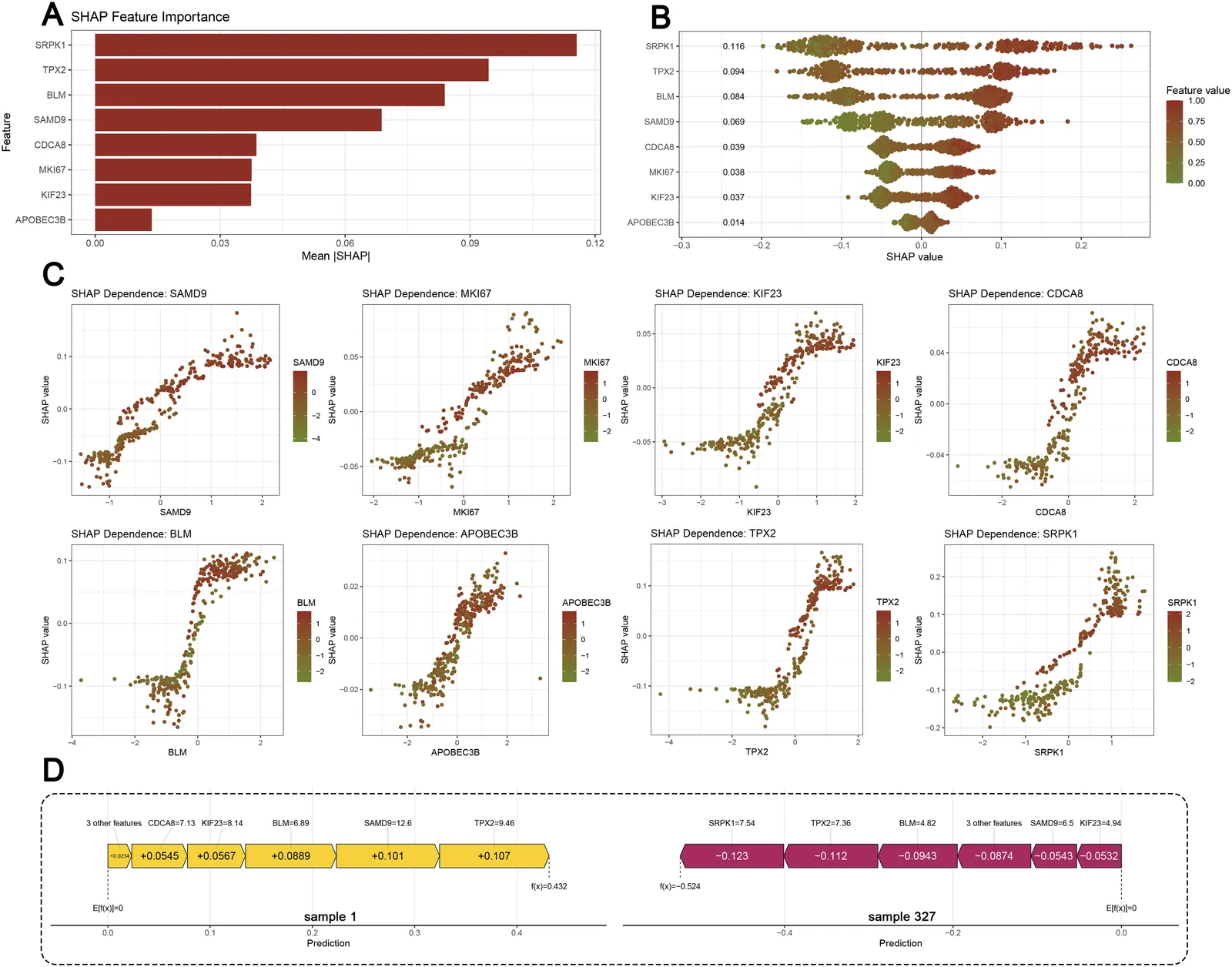
SHAP analysis identifies SRPK1 as the key driver gene in the RRDS model. **(A)** SHAP feature importance bar plot ranking the eight signature genes by their mean absolute SHAP values. SRPK1 shows the highest impact on model output. **(B)** SHAP summary plot displaying the distribution of each feature’s contribution across samples. Each dot represents a sample, colored by normalized expression level (red: high, green: low). For SRPK1, high expression consistently corresponds to positive SHAP values, driving predictions toward psoriasis. **(C)** SHAP dependence plot for SRPK1, illustrating the positive relationship between its expression level and SHAP value, confirming its role as a dominant predictor. Dependence plots for the remaining seven genes are also provided. **(D)** Individual SHAP force plots for two representative samples (sample 1 and sample 327), showing how each gene contributes to shifting the base value toward the final prediction outcome. SHAP, SHapley Additive exPlanations.

### A single-cell atlas of psoriatic skin pinpoints KCs as the primary source of SRPK1 upregulation

Recent advances in scRNA-seq have further enabled high-resolution characterization of cellular heterogeneity and cell-state transitions in complex tissues, thereby complementing bulk transcriptomic analyses in disease research (20). To further validate the tissue-level findings from the bulk RNA-seq analysis at single-cell resolution, we next examined the expression landscape of RRDS genes in the scRNA-seq dataset (GSE173706). Unsupervised clustering and UMAP visualization identified 8 cell types in the skin, including KCs, fibroblasts, smooth muscle cells, melanocytes, endothelial cells, T cells, myeloid cells and nerve cells, with their identities confirmed by top marker gene expression in a dot plot (**Figures 6A, C**). And no significant batch effects were apparent among the diverse samples (**Figure 6B**). Notably, SRPK1 expression was mainly localized within the KCs cluster, with significantly higher expression in KCs from PP versus PN skin (**Figures 6D, E**).

**Figure 6:**
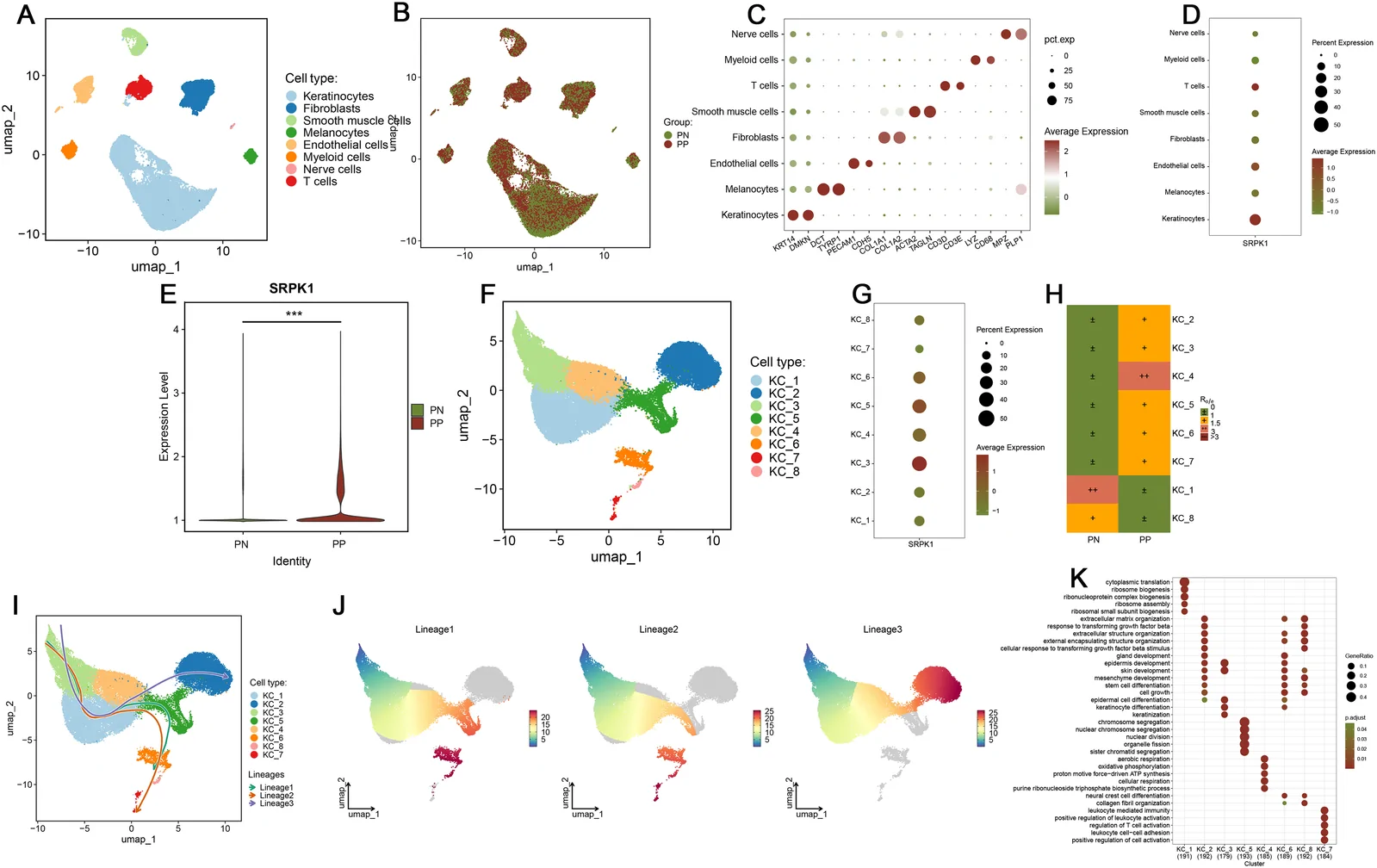
Single-cell analysis reveals specific upregulation of SRPK1 in a pathogenic keratinocyte (KC) subpopulation. **(A)** UMAP visualization of all major cell types identified from the single-cell dataset (GSE173706). **(B)** UMAP plot colored by sample origin (PP: Psoriatic Plaque; PN: Perilesional skin). **(C)** Dot plot showing the expression levels of marker genes for each cell cluster. **(D)** Dot plot showing the expression distribution of SRPK1 across different cell types. **(E)** Violin plot showing the comparison of SRPK1 expression between PP and PN samples. **(F)** UMAP plot of the 8 subclusters identified from re-clustering the KC population. **(G)**​​ Dot plot showing SRPK1 expression across the 8 KC subclusters, with significant enrichment observed in the KC_4 subcluster. **(H)**​​ Heatmap comparing the expression levels of SRPK1 between PP and PN within each of the 8 KC subclusters. SRPK1 expression is significantly elevated in the KC_4 subcluster from PP skin. **(I, J)** Pseudotime trajectory analysis of KC subpopulations, positioning the KC_4 subcluster at a critical branchpoint in the pathological KC differentiation path. **(K)** GO enrichment analysis for marker genes of the 8 KC subclusters. UMAP, Uniform Manifold Approximation and Projection; PP, Psoriatic Plaque; PN, Perilesional; KC, keratinocyte; GO, Gene Ontology. Significance is denoted as *p < 0.05; **p < 0.01; ***p < 0.001.

### SRPK1 is enriched in a specific KC sub-cluster implicated in the pathological differentiation trajectory

To further dissect the role of SRPK1 within KCs, we performed subclustering of the KC population, which revealed eight distinct transcriptional states (KC_1–KC_8). The UMAP visualization displayed these eight distinct KC subpopulations (**Figure 6F**), and dot plots showed that SRPK1 expression was uniquely enriched in the KC_4 subcluster compared to all others (**Figure 6G**). Moreover, a heatmap demonstrated that the expression of SRPK1 was significantly elevated in the KC_4 subcluster from PP compared to PN skin (**Figure 6H**). Pseudotime trajectory analysis positioned this high-SRPK1-expressing KC_4 subcluster at a critical branchpoint along the pathological KC differentiation trajectory, separating the basal/proliferative states from the terminally, aberrantly differentiating states (**Figures 6I, J**). GO enrichment analysis revealed a clear functional specialization among the subclusters, where the KC_4 subcluster was uniquely enriched for terms associated with cellular respiration and cellular energy metabolism, such as ‘Oxidative Phosphorylation’ and ‘Proton Motive Force-Driven ATP Synthesis’ (**Figure 6K**). These results place SRPK1 at a critical cell fate decision point in the pathogenic trajectory of KCs in psoriasis.

### SRPK1 knockdown alleviates keratinocyte hyperproliferation *in vitro*

Given the in silico evidence pointing to SRPK1 as a key driver gene in pathogenic keratinocytes, we next sought to experimentally validate its biological function *in vitro*. We selected the human immortalized keratinocyte cell line, HaCaT, for functional assays. Immunofluorescence staining clearly visualized the expression and subcellular localization of SRPK1 in HaCaT cells, which displayed ubiquitous expression within both the cytoplasm and nucleus, co-localizing with the microtubule network (**Figure 7A**). To investigate the impact of SRPK1 on keratinocyte dynamics, we successfully knocked down SRPK1 expression using specific siRNA. qRT-PCR analysis confirmed a reduction in SRPK1 mRNA levels in the knockdown (KD) group compared to the negative control (NC) group (*P < 0.001*; **Figure 7B**). Since epidermal hyperplasia driven by keratinocyte hyperproliferation is a fundamental hallmark of psoriasis, we performed a CCK-8 assay to assess cell viability. The growth curves spanning 96 hours demonstrated that the proliferation rate of HaCaT cells was significantly impaired following SRPK1 knockdown (*P < 0.001* at 96h; **Figure 7C**). These experimental findings perfectly mirror our single-cell and ML predictions, confirming that SRPK1 is not merely a diagnostic marker, but directly promotes keratinocyte proliferation, thereby driving the progression of psoriasis.

**Figure 7:**
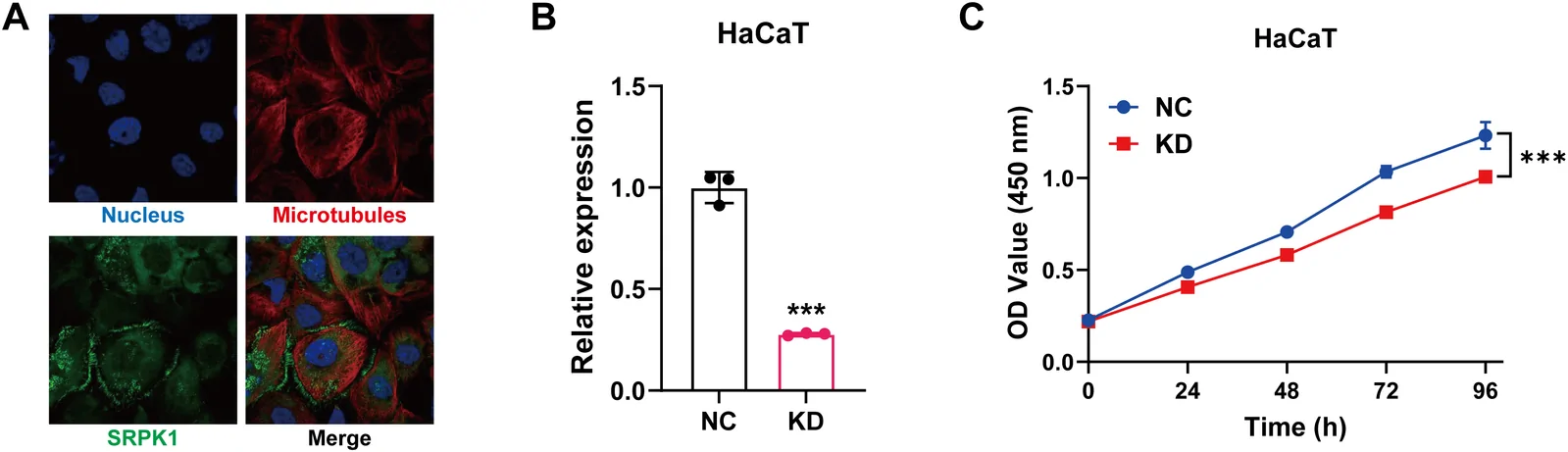
*In vitro* biological validation of SRPK1 in human keratinocytes (HaCaT). **(A)** Representative immunofluorescence image of SRPK1 was retrieved from the Human Protein Atlas (HPA; antibody HPA056486, A-431 cell line). Source: Human Protein Atlas, ENSG00000096063-SRPK1/subcellular. **(B)** Quantitative real-time PCR (qRT-PCR) analysis confirming the knockdown efficiency of SRPK1 in HaCaT cells. Data are presented as relative expression compared to the negative control (NC) group (***P < 0.001). **(C)** CCK-8 proliferation assay over 96 hours demonstrating that SRPK1 knockdown (KD) significantly inhibits the proliferation of HaCaT cells compared to the NC group (*P* < 0.001). Data are presented as mean ± SD of independent biological replicates. Statistical significance was determined by Student’s t-test **(B)** and two-way ANOVA **(C)**. Significance is denoted as *p < 0.05; **p < 0.01; ***p < 0.001.

## Discussion

In recent years, the therapeutic landscape for psoriasis has shifted from traditional therapies to targeted biologics and small-molecule drugs. Despite these advancements, significant clinical challenges persist (21). A primary issue is disease relapse, where lesions frequently recur at their original sites following the cessation of even highly effective biologic therapies (22). This phenomenon is largely attributed to the activated TNF-α/IL-23/IL-17 axis (23) by skin-resident memory T cells (TRM) (24) and KCs possessing inflammatory memory (25). Among these therapies, biologics such as secukinumab have demonstrated marked clinical and molecular efficacy, highlighting the importance of cytokine-directed intervention in modern psoriasis management (2). At the same time, accumulating evidence suggests that psoriasis should be understood as a dynamic immune disorder with substantial cellular plasticity and inflammatory memory, features that may contribute to variable treatment responses, lesion recurrence, and the need for more precise biomarker-guided strategies (3). Consequently, predicting treatment efficacy based on genetic background, immunophenotype, and metabolic profile remains a challenge (26). Against this background, identifying novel molecular dete minants beyond classical immune mediators remains highly relevant.

In this study, we report significantly elevated R-loop activity in psoriatic lesions. Subsequently, we developed and validated an eight-gene diagnostic signature via ML that accurately discriminates between psoriatic and healthy skin. Among these, SRPK1 was identified by SHAP analysis as the core contributor. Single-cell analysis further localized SRPK1 to a pathogenic keratinocyte subpopulation (KC_4) positioned at a critical differentiation node. We subsequently validated these computational predictions through *in vitro* functional experiments. SRPK1 knockdown significantly inhibited keratinocyte proliferation, thereby providing direct biological evidence that SRPK1 functions as a driver of psoriasis-associated keratinocyte hyperproliferation.

A major strength of the present study lies in the integration of bulk transcriptomic modeling with single-cell validation. The RRDS was established from multi-cohort bulk datasets, enabling robust identification of lesion-associated R-loop-related signatures across populations. However, bulk data alone cannot resolve which cellular compartments principally drive these transcriptional changes. By incorporating scRNA-seq analysis, we localized SRPK1 expression to a specific keratinocyte subpopulation, KC_4, and further positioned it at a critical cell-state transition node. This finding provides cellular-context evidence for the bulk-derived model and strengthens the biological plausibility of SRPK1 as a disease driver. Similar integrative approaches have proven informative in psoriasis research, where the combination of transcriptome sequencing and single-cell analysis helped reveal pathogenic pathways, hub genes, and cellular heterogeneity that would be difficult to resolve using bulk data alone (27).

Our RRDS underscores the significantly elevated R-loop activity in psoriatic lesions. Indeed, the dysregulation of R-loop homeostasis is intimately linked to the pathogenesis and progression of various cancers. In LUAD, patients with high R-loop scores exhibited significantly poorer overall survival, with pathway enrichment analysis indicating involvement in cell cycle regulation (28). In melanoma, cells with high R-loop scores drove progression through immune evasion and metabolic reprogramming (29). Similarly, in osteosarcoma, high R-loop scores correlated with poor prognosis due to increased DNA damage (30). To our knowledge, a causal link between R-loop dynamics and psoriasis has not been established. Nevertheless, as psoriasis is characterized by hyperproliferation and inflammation, it is plausible that the associated elevated transcriptional activity leads to R-loop accumulation, mirroring the transcriptional stress observed in cancer cells. The DNA damage and genomic instability caused by R-loop dysregulation offer a potential mechanistic bridge to psoriasis, a disease also characterized by DNA damage. Specifically, oxidative DNA damage, triggered by elevated levels of reactive oxygen species (ROS), acts as a critical factor in psoriasis exacerbation. This ROS-induced damage promotes the release of nuclear DNA fragments into the cytosol, which activates the cGAS-STING innate immune pathway and triggers TNF-α secretion, ​thereby driving​ inflammatory responses (31). Therefore, targeting R-loop-mediated DNA damage may represent a novel therapeutic strategy to mitigate inflammation in psoriasis.

Beyond establishing the RRDS for patient stratification, we identified SRPK1 as a key gene for further investigation. SRPK1 phosphorylates serine-arginine-rich domain-containing proteins, most notably the SR protein family, thereby regulating mRNA alternative splicing (AS) by directing them to the nucleus (32). Additionally, nuclear SRPK1 regulates early spliceosome assembly, influencing the binding of SR proteins to exonic splicing enhancers and thereby modulating AS (33). The role of SRPK1 has been studied in multiple cancer types. In non-small cell lung cancer, SRPK1 promotes tumor progression through the EGFR/AKT pathway, and high expression is significantly associated with poor survival (34). Similar associations with disease progression via NF-κB and PI3K/AKT signaling pathways have been reported in colon cancer (35), gastric cancer (36), endometrial cancer (37), and hepatocellular carcinoma (38). Beyond oncology, SRPK1 has also been implicated in inflammatory diseases. SRPK1 upregulation amplifies the expression of proinflammatory cytokines and promotes cell proliferation via the AKT signaling pathway (39–41). In KCs specifically, SRPK1 has been shown to facilitate HPV16 infection by regulating the activity of the E2 viral replication and transcription factor, and it is essential for late events in the human papillomavirus life cycle (42). These findings together indicate that SRPK1 is involved in both proliferative and inflammatory processes across different disease contexts, positioning it as a candidate biomarker and potential pharmacological target.

In our study, single-cell analysis provided further insight into the specific role of SRPK1 in psoriatic KCs. Our pseudotime analysis positioned the KC_4 subpopulation at a major branchpoint in the differentiation trajectory. The upregulation of SRPK1 specifically at this stage suggests it may act as a regulatory factor at this decision point, potentially driving KCs toward a pathogenic lineage instead of normal differentiation. Based on its established function in regulating alternative splicing, SRPK1 may modulate the processing of genes critical for R-loop dynamics. This regulation could stabilize pathogenic R-loops, promoting a hyperproliferative and pro-inflammatory KC phenotype. This possibility is consistent with the pro-proliferative and pro-inflammatory roles of SRPK1 reported in the studies discussed above (39–41).

To test whether SRPK1 directly affects keratinocyte proliferation, we performed *in vitro* experiments using the human keratinocyte cell line HaCaT. Immunofluorescence staining confirmed the expression of SRPK1 protein in keratinocytes, showing its distribution in both the cytoplasm and nucleus, where it co-localized with the microtubule network. This subcellular localization pattern is consistent with the known dual functions of SRPK1: cytoplasmic phosphorylation of SR proteins and nuclear regulation of spliceosome assembly (32). We then employed siRNA-mediated knockdown to assess the functional consequence of SRPK1 loss. qRT-PCR confirmed efficient suppression of SRPK1 mRNA expression in the knockdown group compared to the negative control. The CCK-8 proliferation assay showed that SRPK1 silencing led to a significant reduction in HaCaT cell proliferation over a 96-hour culture period (*P* < 0.001). Since keratinocyte hyperproliferation is a central pathological feature of psoriatic plaques, this result provides direct evidence that SRPK1 promotes keratinocyte proliferation. These experimental findings are consistent with our single-cell transcriptomic data, which identified SRPK1 upregulation in the pathogenic KC_4 subpopulation, and support the conclusion that SRPK1 contributes to the hyperproliferative phenotype observed in psoriatic epidermis. These results are also in line with previous reports demonstrating that SRPK1 promotes cell proliferation via the AKT signaling pathway in other inflammatory contexts (39–41), suggesting a potentially shared pro-proliferative mechanism in keratinocytes that requires further investigation.

To our knowledge, this research provides a novel assessment​ of ​R-loop related gene​ patterns in ​psoriasis. However, we acknowledge several limitations in our study design. First, although we have provided preliminary *in vitro* evidence supporting a pro-proliferative role for SRPK1 in keratinocytes, our findings regarding the broader RRDS are based primarily on bioinformatic analyses of transcriptomic data, establishing correlation rather than causality between the full R-loop gene signature and psoriasis pathogenesis. Second, while our CCK-8 assay demonstrated a clear effect of SRPK1 on cell proliferation, we did not further investigate the downstream molecular mechanisms, such as the specific signaling pathways like PI3K/AKT, nor did we assess R-loop levels directly upon SRPK1 manipulation. Third, this study was performed based on public databases. Although standardized preprocessing pipelines were implemented, potential batch effects and confounding factors cannot be completely ruled out. Fourth, the immortalized HaCaT cell line is a well-recognized experimental model for biological research on keratinocytes. Nevertheless, it fails to fully recapitulate the *in vivo* physiological characteristics of primary keratinocytes. Restricted by research funding and available experimental conditions, further validation using large-scale human clinical specimens and animal models is absent in the present investigation. Future research will focus on addressing these gaps through: 1) At the cellular level, quantitative assays will be conducted to directly measure R-loop accumulation upon aberrant SRPK1 expression, so as to explore the dynamic alterations of R-loops regulated by SRPK1. Subsequent efforts will focus on elucidating the precise molecular mechanisms and downstream signaling pathways underlying psoriasis progression. 2) In follow-up research, we aim to collect clinical specimens from psoriasis patients, perform multi-omics profiling to establish a specimen biobank. The biological functions of SRPK1 will be further validated in psoriasis animal models including imiquimod-induced mouse models, and the therapeutic potential of SRPK1 inhibition in ameliorating epidermal hyperplasia and inflammatory responses will be investigated. 3) Subject to available experimental resources, the gene-based diagnostic model established in this work will be validated in expanded large-scale clinical cohorts. Correlation analyses will be performed between the SRPK1 protein abundance in lesional skin tissues and clinical severity scores of psoriasis.

## Conclusion

This study provides a comprehensive analysis of R-loop dysregulation in psoriasis, bridging a global pathological phenomenon with a specific molecular driver. We first established that psoriatic lesions are characterized by significantly elevated R-loop activity. Based on this, we developed and rigorously validated a novel eight-gene RRDS which demonstrated exceptional accuracy in distinguishing psoriatic from healthy skin across multiple cohorts. Critically, by integrating ML with advanced interpretability methods, we pinpointed SRPK1 as the pivotal gene within this signature. Single-cell analysis further mapped the function of SRPK1 to a specific pathogenic KC subpopulation (KC_4) at a key differentiation checkpoint, linking its role directly to the disease’s cellular pathology. Furthermore, *in vitro* functional experiments provided direct biological evidence that SRPK1 is robustly expressed in human keratinocytes and that its knockdown significantly suppresses keratinocyte proliferation, thereby validating our computational predictions and confirming SRPK1 as a functional driver of the hyperproliferative phenotype central to psoriasis pathogenesis. Collectively, our findings not only offer a diagnostic tool for psoriasis but also identify SRPK1 as a high-potential therapeutic target. This work highlights the critical role of R-loop regulation in psoriasis pathogenesis and provides a potential target for this complex inflammatory disease.

## Data Availability

The datasets presented in this study can be found in online repositories. The names of the repository/repositories and accession number(s) can be found in the article/**Supplementary Material**.
