# Supplementary material for: Machine learning-derived R-loop-related diagnostic signature for psoriasis: a multi-cohort and single-cell validation study
Source: Front Immunol. 2026 Oct 5;17:1839925. doi: 10.3389/fimmu.2026.1839925 (PMC13677910; doi:10.3389/fimmu.2026.1839925)
Supplement: Supplementary file 1 [file Table1.docx]

**Table S1. Summary of GEO cohorts included in this study**

| Dataset | Total n | Group information | Sample type |
| --- | --- | --- | --- |
| GSE30999 | 170 | NL = 85; LS = 85 | Normal skin and lesional psoriatic skin |
| GSE41664 | 157 | NL = 53; LS = 104 | Normal skin and lesional psoriatic skin |
| GSE13355 | 116 | NL = 58; LS = 58 | Normal skin and lesional psoriatic skin |
| GSE14905 | 58 | NL = 29; LS = 29 | Normal skin and lesional psoriatic skin |
| GSE6710 | 26 | NL = 13; LS = 13 | Normal skin and lesional psoriatic skin |

**Table S3. Primers used for qRT-PCR**

| Gene | Forward primer (5′–3′) | Reverse primer (5′–3′) |
| --- | --- | --- |
| SRPK1 | GCAACAGAATGGCAGCGATC | CTGGCGCTTCTGCTTCTTC |
| β-actin | CACAGAGCCTCGCCTTTGCC | ACCCATGCCCACCATCACG |

**Table S4. siRNA sequences used in this study**

| Name | Sequence (5′–3′) |
| --- | --- |
| si-SRPK1 | GAACAACACATTAGCCAACTT |
